# THE RELATIONSHIP BETWEEN EATING DIFFICULTY AND DEPRESSIVE SYMPTOMS: MODERATING EFFECTS OF EATING AND LIVING ALONE

**DOI:** 10.1093/geroni/igac059.2647

**Published:** 2022-12-20

**Authors:** Junyub Lim, Yeonsoo Shin, Giyeon Kim

**Affiliations:** Chung-Ang University, Seoul, Seoul-t'ukpyolsi, Republic of Korea; Chung-Ang University, Seoul, Seoul-t'ukpyolsi, Republic of Korea; Chung-Ang University, Seoul, Seoul-t'ukpyolsi, Republic of Korea

## Abstract

**Background and Objectives:**

Previous studies report that poor oral health reduces the opportunity for social interaction and increases the risk of psychological distress and that eating alone affects depressive symptoms and its connection differs by living arrangements. The present study aims to investigate the moderating effects of eating alone and living arrangements on the relationship between difficulty eating food due to poor oral health and depressive symptoms among Korean older adults.

**Methods:**

Data were drawn from the 2020 Survey of Living Conditions and Welfare Needs of Korean Older Persons, a nationally representative survey. A total of 9,920 participants aged 65 years and older were included in analyses.

**Results:**

Results from hierarchical regression analyses showed that difficulty eating food was positively correlated with depressive symptoms and eating alone did not moderate the relationship between difficulty eating food and depressive symptoms (b = 1.394, p < .001; b=-0.062, p > 0.05, respectively). However, the effects of eating alone significantly differed by living arrangements (b = 1.663, p < .05). In the case of older adults who live alone, eating alone significantly moderated the relationship between difficulty eating food and depressive symptoms, whereas for those living with others, eating alone did not moderate the relationship. Discussion: Social isolation can be a risk factor that worsens the mental health of older adults who have difficulty eating due to poor oral health. Practical implications for ways to reduce loneliness are discussed.

